# Primary outcomes from Partner2Lose: A randomized controlled trial to evaluate partner involvement on long-term weight loss

**DOI:** 10.21203/rs.3.rs-4001003/v1

**Published:** 2024-03-11

**Authors:** Corrine Voils, Ryan Shaw, Kara Gavin, Scott Hetzel, Megan Lewis, Samantha Pabich, Heather Johnson, Felix Elwert, Lu Mao, Kristen Gray, Alice Yuroff, Katya Garza, William Yancy, Laura Porter

**Affiliations:** University of Wisconsin-Madison School of Medicine & Public Health; Duke University School of Nursing; Medical College of Wisconsin; University of Wisconsin-Madison School of Medicine & Public Health; RTI International; University of Wisconsin-Madison School of Medicine & Public Health; Baptist Health South Florida/Charles E. Schmidt College of Medicine, Florida Atlantic University; University of Wisconsin–Madison; University of Wisconsin School of Medicine and Public Health; University of Washington; University of Wisconsin-Madison School of Medicine & Public Health; University of Wisconsin-Madison School of Medicine & Public Health; Duke University School of Medicine; Duke University School of Medicine

**Keywords:** obesity, randomized controlled trial, behavior therapies, social support

## Abstract

**Background:**

Partner support is associated with better weight loss outcomes in observational studies, but randomized trials show mixed results for including partners. Unclear is whether teaching communication skills to couples will improve weight loss in index participants.

**Purpose:**

To compare the efficacy of a partner-assisted intervention versus participant-only weight management program on long-term weight loss.

**Methods:**

This community-based study took place in Madison, WI. Index participants were eligible if they met obesity guideline criteria to receive weight loss counseling, were aged 74 years or younger, lived with a partner, and had no medical contraindications to weight loss; partners were aged 74 years or younger and not underweight. Couples were randomized 1:1 to a partner-assisted or participant-only intervention. Index participants in both arms received an evidence-based weight management program. In the partner-assisted arm, partners attended half of the intervention sessions, and couples were trained in communication skills. The primary outcome was index participant weight at 24 months, assessed by masked personnel; secondary outcomes were 24-month self-reported caloric intake and average daily steps assessed by an activity tracker. General linear mixed models were used to compare group differences in these outcomes following intent-to-treat principles.

**Results:**

Among couples assigned to partner-assisted (n=115) or participant-only intervention (n=116), most index participants identified as female (67%) and non-Hispanic White (87%). Average baseline age was 47.27 years (SD 11.51 years) and weight was 106.55 kg (SD 19.41 kg). The estimated mean 24-month weight loss was similar in the partner-assisted (2.66 kg) and participant-only arms (2.89 kg) (estimated mean difference, 0.23 kg [95% CI, −1.58, 2.04 kg]). There were no differences in 24-month average daily caloric intake (50 cal [95% CI: −233, 132 cal]) or steps (806 steps [95% CI: −1675, 64 steps]). The percentage of participants reporting an adverse event with at least possible attribution to the intervention did not differ by arm (partner-assisted: 9%, participant-only, 3%, p=0.11).

**Conclusions:**

Partner-assisted and individual weight management interventions led to similar outcomes in index participants.

**Trial registration:**

Clinicaltrials.gov NCT03801174

## Introduction

Excess weight, defined clinically as a body mass index (BMI) of at least 30 kg/m^2^, is associated with cardiovascular disease, hypertension, type 2 diabetes, sleep apnea, disabling osteoarthritis, and some cancers (1–4). Evidence-based weight management programs incorporating behavioral strategies and focusing on dietary change and physical activity can lead to clinically significant weight loss of at least 5% and corresponding improvements in blood pressure, lipids, and blood sugar (5, 6). Many people regain weight following weight loss (7, 8), underscoring the need for effective behavioral strategies to promote long-term weight loss and associated health benefits.

Although lifestyle behaviors occur in a social context, previous weight management interventions have primarily focused on the individual attempting weight loss. Romantic partners have frequent opportunities to eat and engage in physical activity together. Accordingly, eating behaviors and physical activity are correlated within couples, as are weight and weight changes (9). At issue is whether we can intervene on couples to help index participants (i.e., those attempting weight loss) lose and maintain weight and whether this leads to better outcomes than intervening on index participants alone.

Attempts to leverage partner influence on weight management date back to the 1970s (10, 11). Early studies had several significant methodological limitations and did not employ current practices for conducting high-quality randomized trials. Common intervention approaches included using monetary contracts to promote engagement in supportive behaviors or having partners engage in weight management efforts themselves (10, 12–16). When these studies were conducted, there was little conceptual work on social relationships and health to inform the interventions. Since then, there has been more foundational research exploring how couples communicate and influence each other’s health behaviors and outcomes (17, 18). Despite the advances in understanding fundamental processes, few recent trials have tested partner involvement in weight management programs. Recent approaches have not been designed to test the impact of involving versus not involving partners in weight management efforts (19, 20). Studies are needed that apply relational frameworks to intervention development and evaluation.

The conceptual framework for the current study is Lewis’ interdependence model of communal coping and behavior change (18). Communal coping occurs when “one or more individuals perceive a stressor as ‘our’ problem (a social appraisal) vs. ‘my’ or ‘your’ problem (an individualistic appraisal) and activate a process of shared or collaborative coping” (p. 583(21)). Such processes can occur in both “disease-oriented” interventions, in which both members of the couple have the disease and seek treatment, and in “partner-assisted” interventions, in which one member of the dyad supports behavior change in the other (22). Although the goal of partner-assisted interventions is to influence the health outcomes of an index participant, both members of the couple may derive relationship satisfaction, and the partner may reap physical health benefits as well (23–26). Index participants with shared illness appraisals are more likely to communicate with their partner, leading both members of the couple to discuss how to manage the illness; combine efforts, skills, and knowledge to engage in joint problem-solving; and negotiate (27). Observational studies have provided evidence that shared appraisal and collaboration are associated with health behaviors and psychosocial outcomes (28). Unclear is how to intervene to increase these two functions.

One possible approach to increasing communal coping is to apply principles of cognitive behavioral couples therapy (CBCT) (29) to enhance communication skills for sharing thoughts and feelings and joint problem solving. Applications of CBCT have reduced relationship distress and improved relationship functioning across a range of psychological disorders (22, 30). In physical health contexts such as cancer, arthritis, chronic pain, cardiovascular disease, and diabetes, where treatment targets have varied, CBCT strategies have led to mixed findings, suggesting more research is needed (30).

In the present investigation, we applied CBCT strategies to couples involving an index participant attempting weight loss and a cohabiting romantic partner. We evaluated the efficacy of the partner-assisted intervention compared to participant-only intervention. Our hypotheses were that the partner-assisted intervention would lead to greater weight loss and physical activity and reduced caloric intake at 24 months than the participant-only intervention. We also evaluated the impact of the intervention on interdependence constructs and whether these constructs mediated intervention effects.

## Methods

### Design

The study was a two-group, parallel, randomized controlled trial. Couples comprising an index participant for whom obesity criteria guidelines recommend weight loss (4) and a cohabiting partner were randomly assigned 1:1 to the participant-only or partner-assisted arm. Randomization was stratified into eight strata by three variables likely to be associated with weight loss: index participant sex and baseline BMI (< 35 kg/m^2^ vs. ≥35 kg/m^2^) and partner BMI (< 27 kg/m^2^ vs. ≥27 kg/m^2^) (31, 32). The study was conducted in five cohorts of 38–50 couples. The primary outcome was weight measured at 24 months; secondary outcomes included estimated daily caloric intake and steps at 24 months. The first participant provided informed consent on January 15, 2019; the last date of follow-up was March 20, 2023. The study protocol was approved by the University of Wisconsin Health Sciences institutional Review Board. The trial was registered at clinicaltrials.gov (NCT03801174), where the full protocol can be accessed.

### Setting

The first three cohorts were recruited from Madison, WI and the surrounding community; the fourth and fifth cohorts were recruited from across the state of Wisconsin due to the ability to deliver the intervention and collect outcomes virtually following onset of the COVID-19 pandemic. Group weight loss classes were delivered in person in community spaces (e.g., churches, community centers) prior to March 2020 and by secure videoconferencing thereafter. Individual counseling was delivered by telephone throughout the study.

### Eligibility and Recruitment

Eligibility criteria were reported previously (33). Index participant criteria were aged 18 to 74 years; BMI of 27–29.9 kg/m^2^ with at least one obesity-related comorbidity or BMI of at least 30 kg/m^2^; cohabitation and daily contact with a spouse or romantic partner; English speaking; and possession of a smart phone and e-mail address. In the first three cohorts, participants were required to have blood pressure < 140/90 mmHg; this criterion was removed during the pandemic because it required physical contact with study staff and elevated blood pressure was not deemed a safety concern by the study physicians or data safety monitoring board. Exclusion criteria included: recent weight loss; participation in a program focusing on lifestyle changes; current use of weight loss medications; history of, or plans for, bariatric procedure; severely impaired hearing; current treatment for cancer besides skin cancer; use of diabetes medications that increase risk for hypoglycemia; pregnant, breastfeeding, or planning to become pregnant; and current medications or chronic health problems that would limit the ability to participate (e.g., severe kidney disease). Partner inclusion criteria included age 18 or older and possession of a smart phone and e-mail address separate from the index participant. Partner exclusion criteria included: underweight BMI (< 18.5 kg/m^2^); severely impaired hearing; and health condition that would limit one’s ability to provide support. In each cohort, two groups were formed for each arm. Each group met on a different day and time, and this schedule changed for each cohort. To be eligible, couples had to be available for at least one group meeting time for each arm.

Each cohort was recruited over eight weeks. We placed advertisements in the community on bulletin boards (e.g., coffee shops, grocery stores) and sent an e-mail to university employees. To enhance recruitment of Black and Hispanic persons and of men, we used several methods: placed advertisements on websites addressing those populations; wrote several health-related articles for a website serving the local Hispanic population; sent recruitment letters to individuals meeting BMI criteria who were identified as Black or Hispanic persons via electronic health record data in local family and internal medicine practices; and placed advertisements aimed at men in Reddit.

All advertisements and recruitment letters directed people to a screening website, where initial eligibility for index participants and partners was assessed. Couples passing this step completed additional telephone screening and were scheduled for a baseline visit, which occurred in person for cohorts 1–3 and virtually for cohorts 4 and 5. At the baseline visit, final eligibility was determined, and eligible persons provided written informed consent, completed baseline measures, and then were randomized.

### Randomization and Interventions

Couples within strata were randomly assigned in block sizes of four or six. A statistician generated the randomization scheme and uploaded it into Research Electronic Data Capture (REDCap), a secure online software platform for data capture and storage (34, 35). At the baseline visit, study staff accessed the randomization assignment in REDCap following consent and completion of baseline measures. The assignment was displayed to study staff and consented couples as a class date and time instead of group assignment to allow masking for outcome assessments. The principal investigator revealed arm assignment at the first group class, which was attended only by index participants. Index participants in the participant-only arm were informed that their partners and they could attend two communication skills classes following completion of the study.

Both arms received an evidence-based weight management program based on prior research involving a 6-month weight loss phase followed by a 12-month weight loss maintenance phase (36). The final 6 months involved no further intervention contact to yield data on outcome durability. The weight loss phase involved 13 group classes every other week addressing nutrition and behavioral strategies and including physical activity demonstration. At baseline, all index participants received an intervention manual and a Fitbit^®^ physical activity tracker (37). In every class, participants set a SMART (specific, measurable, actionable, relevant, and time-bound) goal related to a menu of several topics (e.g., for meal planning, options included: serving sizes, meals and snacks, calorie meal plan, and fiber). Between classes, participants in both arms received three text messages per week. These messages provided reminders of their SMART goal topic and key messages from the class (both arms), as well as support plans (participants in the partner-assisted arm) (38).

The maintenance period involved three group classes and nine telephone calls focusing on four maintenance strategies (36, 39): satisfaction with weight loss outcomes, self-monitoring, relapse prevention, and social support. The calls were delivered monthly for six months and then every other month. In months 7–9, participants received two text messages per week with maintenance skill content. In months 10–12, text message frequency decreased to once per week, and, from months 13–18, it reduced to once every two weeks. To minimize burden and provide an opportunity to discuss challenges without the partner present, we mandated that partners attended half of the group sessions (33).

All classes and telephone calls in the partner-assisted arm included extra content on communication skills derived from CBCT. In the first two classes, the interventionist introduced the skills of sharing thoughts and feelings and joint problem-solving, respectively. In subsequent classes, couples were given five-minute breakouts to apply those skills to the class topic. Additionally, index participants shared their SMART goal with their partner, and couples worked together to devise a support plan from a provided list (i.e., do it together, provide gentle reminders, praise your partner, remember the long game, check in with your partner, be mindful of how your choices affect your partner’s goals, and talk with your partner to develop a support plan at home). The support plan was reported to study staff so that a tailored text message reminding them of their support plan could be sent the following week. Partners in the partner-assisted arm received text messages at the same frequency as index participants, which included didactic content, social support tips, and reminders of their support plans.

The group sessions and telephone calls were delivered by one of two registered dieticians (RDs). Training included a review of prior literature on CBCT, behavioral weight loss, and initiation versus maintenance skills (39). The RDs delivered dry runs of the group sessions with the principal investigator and research staff. They also practiced calls with mock participants. All classes and calls were audio recorded. We created fidelity checklists for the classes and calls that included nutrition content and behavioral skills. In cohort 1, the principal investigator and a study physician attended all group classes to monitor fidelity; in cohorts 2–5, a clinical psychology co-investigator or doctoral-level scientist reviewed audio recordings for 75% of classes (all partner-assisted and half of participant-only). These individuals also reviewed recordings for 10% of all maintenance calls, completing fidelity checklists. Calls were both randomly selected by the team and suggested by the RDs for review. The intervention team made up of the principal investigator, two co-investigators, and RDs met every other week throughout the study to listen to additional calls, discuss challenges, and provide feedback.

### Outcomes and Follow-up

Couples were scheduled for assessments at months 6, 12, 18, and 24. Measurements were conducted by staff masked to group assignment. Participants and partners each received $40 for assessments at months 6, 12, and 18. Cohorts 1–4 received $60 at month 24, and cohort 5 received $70 to enhance retention.

Weight taken at the first in-person group class or within one week prior to the first virtual group class served as baseline. Participants were asked to weigh in light clothing and no shoes. Height was assessed at the eligibility visit with a portable stadiometer prior to the pandemic and was self-reported thereafter. Initially, all weights were collected by study staff using a calibrated Tanita^®^ scale; after the onset of the pandemic, all participants received a bathroom scale by mail. Depending on current regulations and participant comfort with in-person contact, participants could be weighed in person or submit by e-mail a photo of their feet and weight on the scale. A team member reviewed all photos and entered the verified weights into REDCap.

Dietary intake was assessed at each time point with the Automated Self-Administered Dietary Assessment 24-hour Dietary Assessment Tool (ASA24) with a 6-month recall period (40, 41). Participants received an e-mail or text message prompting them to enter one weekday and one weekend day during the 2-week assessment period. Those who did not enter data within 24 hours received a text message and e-mail prompting them to complete the recall, along with a video created by our team to demonstrate how to use the ASA24.

Steps were measured with Fitbit wrist-worn activity trackers (42). Participants received a device at baseline and reminders to wear it for 7 consecutive days during each assessment window. We calculated the average steps per day during the 7-day timeframe at each time point. Days in which < 1000 steps were recorded were removed for analyses, and participants had to have data for at least four of the seven days to be included.

We assessed baseline relationship closeness with the Unidimensional Relationship Closeness Scale (43). We assessed interdependence constructs and social support as potential mediators at baseline and every three months up to month 21. Transformation of motivation was measured via Aron’s inclusion-of-other-in-self figure, one each for relationship, weight loss, diet, and physical activity, scored from 1–7 with higher scores indicating greater overlap of other and self. Because there are no published, validated measures, the study team created items to assess couple efficacy (0 = not at all confident to 10 = very confident), outcome efficacy (0 = not at all confident to 10 = very confident), and communal coping (0 = never, 1 = sometimes, 2 = frequently, 3 = often, 4 = very often), each with 5 items for healthy eating and 5 for physical activity (33). Social support for healthy eating was measured with 6 items, sabotage for healthy eating with 3 items, social support for physical activity with 6 items, and sabotage for physical activity with 6 items (44). Per recommendations of Kiernan, social support and sabotage were scored 1 = almost never, 2 = rarely, 3 = sometimes, 4 = often, and 5 = almost always (45).

Demographics assessed during the screening visit included self-reported race, ethnicity, sex assigned at birth (used for stratification), gender identity, marital status, education, work status, financial stress, health insurance coverage, tobacco use, and number of previous weight loss attempts. Age was taken from the website screener.

Participants reported adverse events (AEs) to a team member during intervention contacts or outcome assessments. A study physician rated each AE according to severity, relatedness, and expectedness. All events were categorized according to the Common Terminology Criteria for Adverse Events version 5.0 (46). The institutional data safety monitoring committee reviewed all events in annual meetings.

### Statistical Analysis

Descriptive statistics of participant characteristics were calculated overall and by treatment group, with quantitative variables summarized by mean (standard deviation; SD) or median (inter-quartile range; IQR) and categorical variables by n (%). All outcomes were analyzed according to intent-to-treat principles. Weights measured at months 0, 6, 12, 18, and 24 were modeled by the linear mixed effects (LME) model against the discrete time points and interactions with the treatment group and patient as a random effect, where the baseline group means were constrained to be the same owing to randomization.^24^ Fixed covariates in the LME model included randomization strata and recruitment cohort. Weight loss at 24 months from baseline was calculated by contrasts of regression coefficients in the model. We used similar models for testing the effect of treatment on secondary outcomes. To enable comparisons with other studies, we calculated the percentage of participants achieving at least 5% weight loss in each arm at each time point without conducting inferential tests. Missing data were imputed using Multiple Imputations by Chained Equations (47).

Pre-planned causal mediation analysis (48, 49) was used to estimate the extent to which the intervention effect at month 6 was jointly mediated by the set of communal coping constructs (transformation of motivation, couple efficacy, outcome efficacy, use of communal coping, and social support) measured at 3 months. Specifically, we estimated two natural effects models for the natural direct and the natural indirect effects of the intervention via communal coping—one for communal coping with respect to diet, and another with respect to physical activity. A significance level of p ≤ 0.05 was used in all analyses. All analyses were performed in R version 4.0.2 (R Foundation for Statistical Computing, Vienna, Austria).

Our primary hypothesis was that participant weight loss would be at least 2.5 kg lower at 24 months in the partner-assisted than participant-only arm. This effect size is considered clinically meaningful to providers and is like that of other weight management studies (4). For the power analysis, we assumed a common standard deviation of 19.9 kg, an intraclass correlation among group members of 0.01, and a correlation between baseline and month 24 weight measurements of 0.96 based on a previous study (36). With a type I error rate of 5%, power of 80%, and a dropout rate of 20%, the calculated sample size was 230 total (115 per arm).

## Results

### Participants

**Figure 1:**
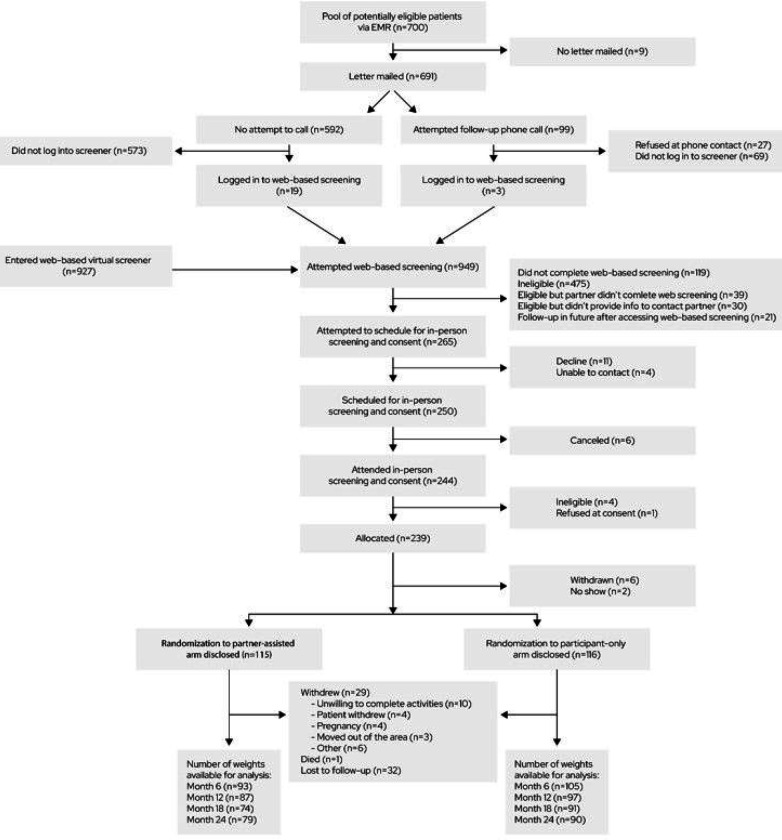
CONSORT flow diagram

As shown in Fig. 1, 927 individuals started screening in response to advertisements, and 22 started the web-based screener after receiving a recruitment letter. Of those, 239 were eligible and provided consent, and 231 (n = 115 partner-assisted, n = 116 participant-only) provided a baseline weight and were randomized. Of the 231 partnerships represented, 61.90% were female index participant/male partner and 31.17% were male/female, with 87.45% of couples married and the remainder domestic partners (Table 1). The majority identified as female and as non-Hispanic White. The average baseline age was 47.27 years. Nearly all participants had more than a high school education and insurance through an employer, three-fourths were employed full-time, and few indicated financial hardship. Index participants’ average baseline weight was 106.55 kg (SD 19.41 kg) and BMI was 37.14 kg/m^2^ (SD 6.43 kg/m^2^). Their average estimated baseline daily caloric intake was 2143 kcal and number of daily steps estimated by Fitbit was 8114.

### Primary and Secondary Outcomes

**Figure 2:**
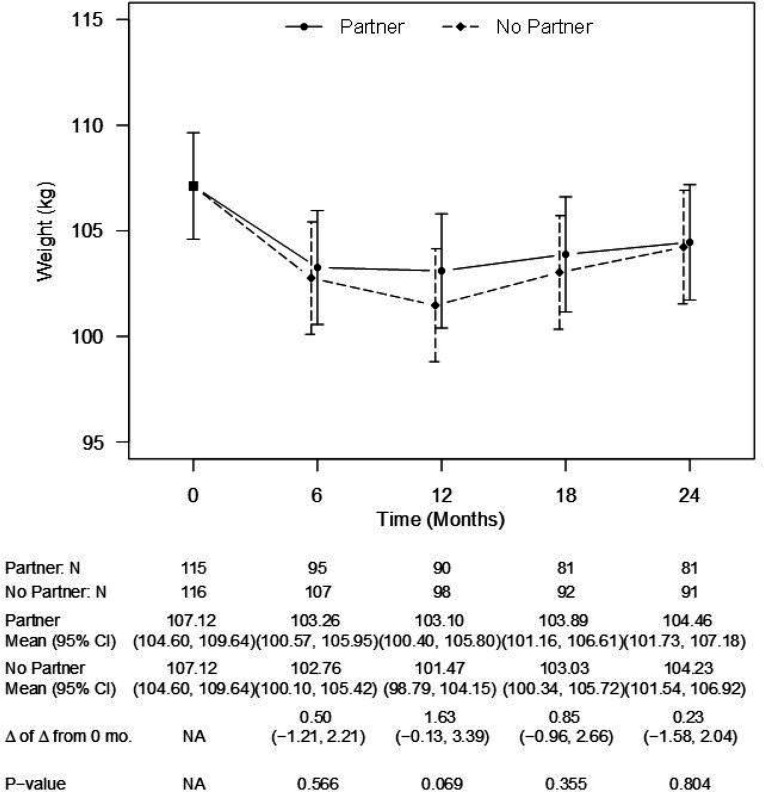
Model-estimated weights, differences in weights, and associated 95% CIs, by treatment group and time point

Across 24 months, the average estimated weight loss was 2.66 kg for participants in the partner-assisted arm and 2.89 kg for those in the participant-only arm (Fig. 2). The between-groups difference was not significant at 24 months (estimated difference 0.23 kg [95% CI: −1.58, 2.04 kg], p = 0.80), nor any of the interim time points (all p > .06). A similar pattern emerged in a sensitivity analysis involving multiple imputation (estimated mean difference at 24 months 0.7 kg [CI: −1.67, 3.07 kg], p = 0.56). The percentages of participants achieving at least 5% weight loss from baseline in the partner-assisted and participant-only arms were 33.68% and 35.51% at 6 months, 38.89% and 46.94% at 12 months, 34.57% and 40.22% at 18 months, and 33.33% and 31.87% at 24 months.

**Figure 3:**
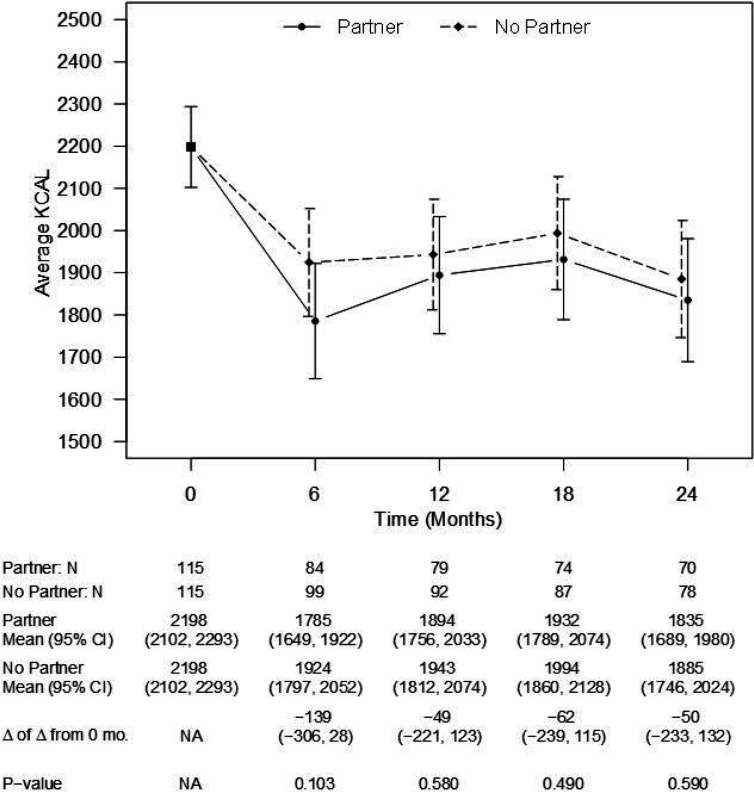
Model-estimated daily caloric intake, differences in caloric intake, and associated 95% CIs, by treatment group and time point

**Figure 4:**
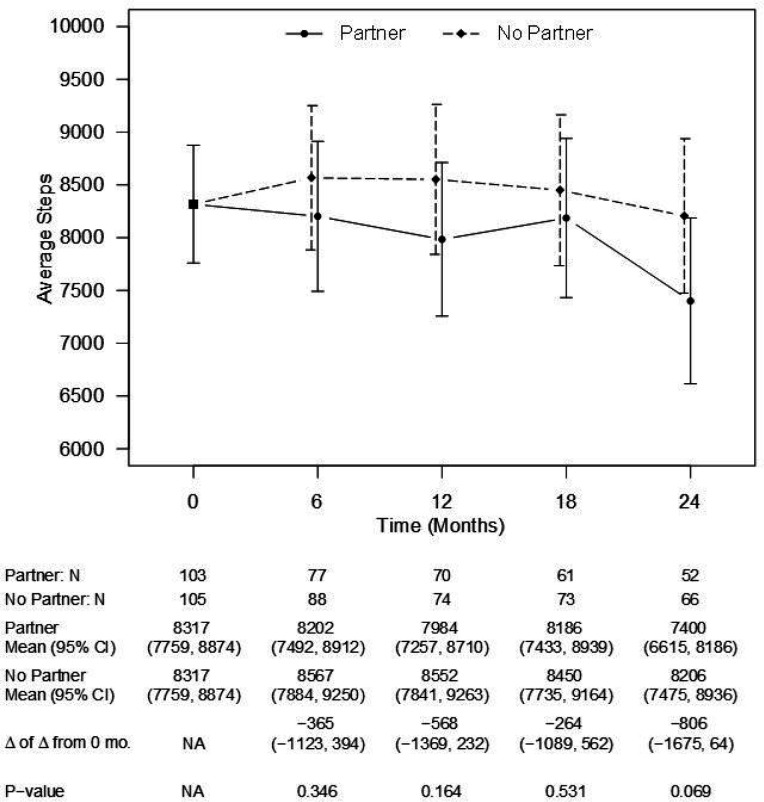
Model-estimated daily steps, differences in steps, and associated 95% CIs, by treatment group and time point

Like weight, there were no between-group differences in estimated daily caloric intake at any time point (Fig. 3). The estimated between-group difference at 24 months was 50 cal (CI: −233, 132 cal, p = 0.59). There were also no differences in estimated daily steps at any time point (Fig. 4). The estimated between-group difference at 24 months was 806 steps (CI: −1675, 64 steps, p = 0.07).

### Mediation via Interdependence Constructs

**Figure 5:**
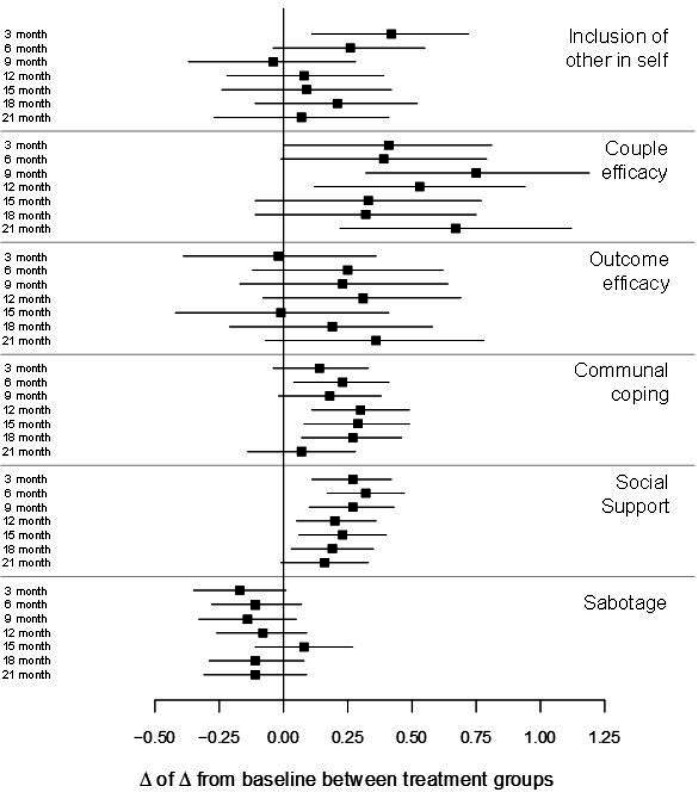
Between-arm differences in interdependence constructs related to dietary change

Between-arm differences in interdependence constructs related to dietary change are shown in Fig. 5 (for values, see Supplement 1). Communal coping, couple efficacy, and social support were significantly greater in the partner-assisted arm at most time points, whereas inclusion of other in self was greater only at 3 months, and outcome efficacy and sabotage did not differ at any time point. Although the intervention improved some constructs, these improvements did not mediate the treatment effect: The estimated natural direct effect was 0.04 (95% CI: −5.64, 5.73), p = 0.99; natural indirect effect was − 0.05 (95% CI: −2.35, 2.24), p = 0.96; and total effect was − 0.01 (95% CI: −5.17, 5.14), p > 0.99.

**Figure 6:**
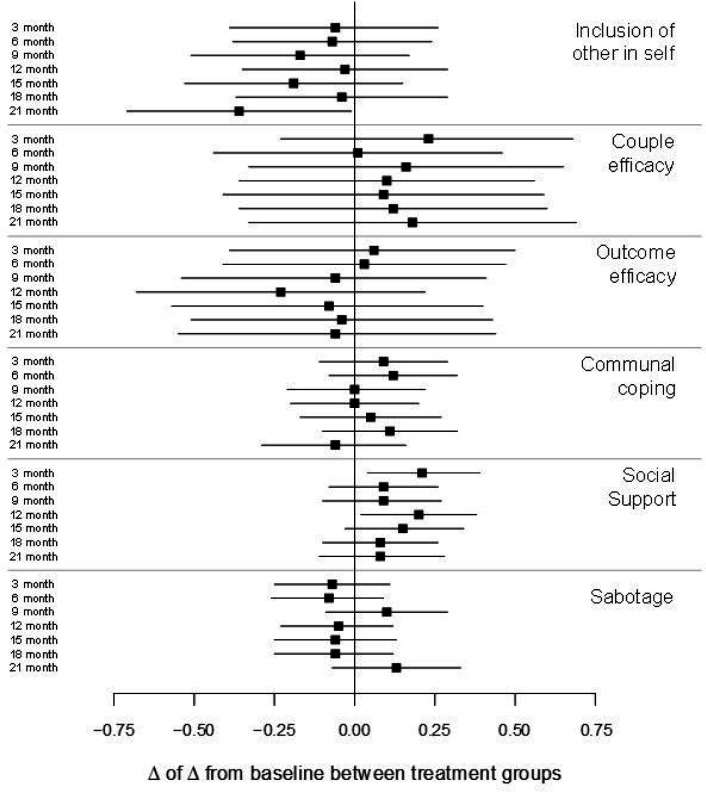
Between-arm differences in interdependence constructs related to physical activity

Between-arm differences in the potential mediators for physical activity are shown in Fig. 6 (Supplement 2). There were no consistent differences in any construct at any time point and no evidence of mediation. The estimated natural direct effect was 0.22 (95% CI: −5.00, 5.43), p = 0.94; natural indirect effect was 0.14 (95% CI: −1.47, 1.75), p = 0.86; and total effect was 0.36 (95% CI: −4.89, 5.60), p = 0.89.

### Intervention Adherence and Retention

In the partner-assisted arm, index participants attended a median of 13 (of 16) classes (range 1 to 16, interquartile range [IQR] = 7,14.5) and a median of 8 (of 9) calls (range 0 to 9, IQR = 0,9); in the participant-only arm, they attended a median of 12 classes (range 1 to 16, IQR = 9,14) and a median of 7 calls (range 0 to 9, IQR = 2,9). The between-arm difference in classes attended was not significant, p = 0.68, nor was the difference in calls received, p = 0.94. Partners attended a median of 6 (of 6) planned classes (range 0 to 9, IQR = 4,8) and a median of 4 maintenance (of 5) phone calls (range 0 to 5, IQR = 3,5). The average duration of group classes was 74.1 (SD = 17.1) minutes in the partner-assisted arm when participants attended alone and 79.7 (SD = 18.3) minutes when they attended with their partner, whereas it was 69.3 (SD = 13.6) minutes in the participant-only arm. The average duration of counseling telephone calls was 22.9 (SD = 9.6) minutes in the partner-assisted arm when participants attended alone and 31.6 (SD = 13.2) minutes when they attended with their partner, whereas it was 25.7 (SD = 9.5) minutes in the participant-only arm. The retention rates at 24 months in the partner-assisted and participant-only arms were 70% and 78% for weight, 61% and 67% for caloric intake, and 45% and 57% for daily steps measurements, respectively. No crossover of assignments occurred. Sixteen couples in the participant-only arm attended the two virtually delivered communication skills classes following completion of the 24-month outcome assessment.

### Adverse Events

A similar percentage of participants in each arm reported any AE (partner-assisted arm: 50%; participant-only: 47%), p = 0.69, or an SAE (3% in each arm, p = 0.99). There was one death in the partner-assisted arm, unrelated to the intervention. The percentage of participants reporting an AE with at least possible attribution to the intervention did not differ by arm (partner-assisted arm: 9%; participant-only arm 3%, p = 0.11). Out of the 214 reported (AEs), most were mild or moderate (partner-assisted arm: 81%; participant-only: 78%).

## Discussion

This randomized controlled trial tested the impact of a partner-assisted approach to promote long-term weight loss among people for whom behavioral weight management is recommended (4). The partner-assisted intervention was designed to enhance both shared appraisal and collaboration to enable partners to support index participants in lifestyle change. Although the intervention increased participant perceptions of communal coping, couple efficacy, and social support for diet, it did not differentially affect the same variables for physical activity, nor did it affect the primary or secondary outcomes relative intervening on index participants alone.

Our findings are largely consistent with recently published partner-assisted interventions applied to behavior change. In one trial (50), patients with uncontrolled type 2 diabetes and their partners were randomized to telephone-based diabetes education, individual calls, or couples’ calls. In the couples’ calls, the dietitian encouraged couples to apply collaborative problem-solving and communication management to dietary change, activity, medication adherence, and glucose testing. There was no benefit of the couples’ intervention on blood glucose, BMI, waist circumference, blood pressure, diabetes self-efficacy, nor depressive symptoms. However, the patients in partner-assisted arm had significantly lower diabetes distress scores at 12 months and greater treatment satisfaction compared to the education control group. This trial differs from ours in that the intervention was delivered to individuals rather than in groups, and patients were managing a chronic disease with greater self-management requirements compared to obesity. In the Partners for Life intervention (51), married patients with a diagnosis of coronary artery disease or a cardiac event or procedure were randomized to partner-assisted or participant-only intervention delivered in groups to improve dietary intake, physical activity, and medication adherence. Couples in the partner-assisted arm received training in CBCT communication skills and practiced them in breakout sessions. Patients in the partner-assisted arm experienced greater increases in physical activity but not dietary intake or medication adherence. This study differs from ours because participants had an acute health stressor (compared to a chronic disease), the intervention was taught by a therapist (compared to a dietitian), spouses participated in all sessions (compared to half), the groups involved up to five couples (compared to up to 16), and the therapists were able to observe and provide feedback on communication processes (ours were not). Taken together, these trials, which carefully isolated the impact of couples’ communication training, have found little overall benefit on clinical outcomes.

Several possible explanations can be provided for our null findings. First, the dose of partner involvement may have been too small to have a meaningful impact. Partners were required to attend half of the sessions, which were < 10 minutes longer compared to participant-only sessions. More time may be needed to practice implementing communication skills. Relatedly, with group-based intervention, the interventionist could not observe and provide feedback to couples as they practiced implementing the skills during breakouts in group classes. Many previous interventions have involved counseling between an individual therapist and couple, which may be required to enhance outcomes (22, 52, 53).

Another possible explanation for the null effect is that improvement in communal coping and social support do not lead to improvements in weight loss over and above the effects of behavior change strategies such as self-monitoring, setting graded tasks, review of behavioral goals, and barrier identification and problem solving (54). Our intervention involved all these strategies and isolated the impact of improving partner interactions. Another explanation is that couples in both arms were generally supportive at baseline, reducing the possibility of observing an effect. Finally, interventions aimed at increasing communication and collaboration skills may not be an effective method to increase communal coping in the context of obesity as they are in other contexts such as pain management, cancer, and the end of life (55).

The findings must be interpreted in the context of several limitations. One is that, despite our best efforts to recruit a diverse sample, most participants identified as non-Hispanic White and female and were middle-aged and educated, thus limiting generalizability. Yet, a strength is that we were able to recruit people living outside the metropolitan area where the university is located after switching to virtual delivery. Additional efforts are needed to improve representation and belonging of populations not well-represented in weight management studies. Another limitation is that dietary intake was self-reported, and we only captured daily steps rather than other types of exercise. Moreover, we had lower retention rates for the ASA24 and step data compared to our primary outcome of weight. Another limitation is that our retention rate for the primary outcome was lower than we assumed in our power calculation despite offering multiple methods for participants to provide data. Using a cellular-enabled scale for data capture and transmission may be a more effective approach to enhancing retention (56–58). Finally, although none of our measured variables was associated with retention, retention may have been related to unmeasured variables associated with the experience of the pandemic, such as stress or caregiving responsibilities.

Our study also has several strengths. It is one of few fully powered trials to test the efficacy of a theory-based, partner-assisted weight loss intervention. We compared the intervention to a participant-only intervention to isolate the effects of the partner components. In contrast to many weight management studies, which focus on initial weight loss and are one year or less in duration, our intervention additionally addressed maintenance and measured effects at two years. Our average trial participant lost 3% of their body weight and kept it off to a large degree across the study. This is notable given that many people gained weight during the pandemic (59–61). Additional strengths include high intervention adherence by both index participants and partners and testing of theory-based mediators.

## Conclusions

Partner2Lose is an important attempt to isolate and evaluate the impact of systematically involving partners in a weight loss program and teaching couples communication skills. To build on this work, future studies should consider strategies to strengthen partner engagement and couple communal coping, such as increasing the dose and conducting sessions with individual dyads rather than in groups. Identifying couples who are most likely to benefit from this approach (e.g., couples with worse relationship functioning/communal coping at baseline, or couples who are both attempting to lose weight) may also be beneficial. Finding ways to enhance communal coping may hinge on other intervention approaches that promote shared appraisals between partners to manage weight loss (21, 28).

## Figures and Tables

**Table 1:** Characteristics of participants, overall and by treatment group^a^

Characteristic	Participant			Partner		
	Overall (n = 231)	Partner-assisted (n = 115)	Participant-only (n = 116)	Overall (n = 231)	Partner-assisted (n = 115)	Participant-only (n = 116)
Partnership identities (Participant:Partner)^b^						
Female:Male	143 (61.90%)	72 (62.61%)	71 (61.21%)			
Male:Female	72 (31.17%)	35 (30.43%)	37 (31.90%)			
Female:Female	11 (4.76%)	4 (3.48%)	7 (6.03%)			
Male:Male	2 (0.87%)	2 (1.74%)	0 (0.0%)			
Female:Multi-gender	1 (0.43%)	0 (0.00%)	1 (0.86%)			
Genderqueer:Genderqueer	1 (0.43%)	1 (0.87%)	0 (0.00%)			
Multi-gender:Female	1 (0.43%)	1 (0.87%)	0 (0.00%)			
Married partnership	202 (87.45%)	97 (84.35%)	105 (90.52%)			
Assigned female sex at birth^c^	157 (67.97%)	78 (67.83%)	79 (68.10%)	87 (37.66%)	42 (36.52%)	45 (38.79%)
Gender identity						
Female	155 (67.10%)	76 (66.09%)	79 (68.10%)	84 (36.36%)	40 (34.78%)	44 (37.93%)
Male	74 (32.03%)	37 (32.17%)	37 (31.90%)	145 (62.77%)	74 (64.35%)	71 (61.21%)
Genderqueer	1 (0.43%)	1 (0.87%)	0 (0.00%)	1 (0.43%)	1 (0.87%)	0 (0.0%)
Multi-gender	1 (0.43%)	1 (0.87%)	0 (0.00%)	1 (0.43%)	0 (0.00%)	1 (0.86%)
Age	47.27 (11.51)	47.31 (11.31)	47.24 (11.76)	48.25 (12.11)	48.43 (12.48)	48.07 (11.78)
Not Hispanic/Latino	220 (95.65%)	108 (94.74%)	112 (96.55%)	218 (95.20%)	109 (95.61%)	109 (94.78%)
Race						
White	198 (86.84%)	97 (85.09%)	101 (88.60%)	198 (88.00%)	99 (89.19%)	99 (86.84%)
Black or African American	8 (3.51%)	4 (3.51%)	4 (3.51%)	12 (5.33%)	9 (8.11%)	3 (2.63%)
Asian	12 (5.26%)	6 (5.26%)	6 (5.26%)	10 (4.44%)	2 (1.80%)	8 (7.02%)
American Indian or Alaska Native	3 (1.32%)	2 (1.75%)	1 (0.88%)	1 (0.44%)	0 (0.00%)	1 (0.88%)
Multiracial	7 (3.07%)	5 (4.39%)	2 (1.75%)	4 (1.78%)	1 (0.90%)	3 (2.63%)
High school graduate or less	8 (3.46%)	6 (5.22%)	2 (1.72%)	10 (4.35%)	8 (6.96%)	2 (1.74%)
Employed full-time	176 (76.52%)	88 (77.19%)	88 (75.86%)	162 (70.43%)	85 (74.56%)	77 (66.38%)
Financial status						
Poor/Just getting along	22 (9.52%)	10 (8.70%)	12 (10.34%)	26 (11.26%)	17 (14.78%)	9 (7.76%)
Prosperous	11 (4.76%)	7 (6.09%)	4 (3.45%)	13 (5.63%)	5 (4.35%)	8 (6.90%)
Reasonably comfortable	119 (51.52%)	62 (53.91%)	57 (49.14%)	119 (51.52%)	56 (48.70%)	63 (54.31%)
Very comfortable	79 (34.20%)	36 (31.30%)	43 (37.07%)	73 (31.60%)	37 (32.17%)	36 (31.03%)
Financial situation						
Difficulty paying bills	2 (0.87%)	1 (0.87%)	1 (0.86%)	0 (0.00%)	0 (0.00%)	0 (0.00%)
Enough to pay bills after cut back	12 (5.19%)	4 (3.48%)	8 (6.90%)	12 (5.22%)	10 (8.70%)	2 (1.74 %)
Little to spare for special things	62 (26.84%)	37 (32.17%)	25 (21.55%)	68 (29.57%)	35 (30.43%)	33 (28.70%)
Enough for special things	155 (67.10%)	73 (63.48%)	82 (70.69%)	150 (65.22%)	70 (60.87%)	80 (69.57%)
Health insurance						
Employer	207 (90.00%)	102 (88.70%)	105 (91.30%)	200 (88.11%)	101 (89.38%)	99 (86.84%)
Self-purchased	9 (3.91%)	4 (3.48%)	5 (4.35%)	14 (6.17%)	6 (5.31%)	8 (7.02%)
Medicare	18 (7.83%)	9 (7.83%)	9 (7.83%)	26 (11.45%)	12 (10.62%)	14 (12.28%)
Medicaid	5 (2.17%)	3 (2.61%)	2 (1.74%)	3 (1.32%)	2 (1.77%)	1 (0.88%)
Military	3 (1.30%)	2 (1.74%)	1 (0.87%)	2 (0.88%)	1 (0.88%)	1 (0.88%)
VA	1 (0.43%)	0 (0.00%)	1 (0.87%)	4 (1.76%)	1 (0.88%)	3 (2.63%)
Currently use nicotine	8 (3.48%)	2 (1.75%)	6 (5.17%)	13 (5.63%)	10 (8.70%)	3 (2.59%)
Attempted weight loss previously	218 (94.37%)	110 (95.65%)	108 (93.10%)	175 (76.09%)	86 (74.78%)	89 (77.39%)
Number of attempts, median (IQR)	3.0 (2.00–5.00)	3.00 (2.005.75)	3.00 (2.00–5.00)	3.00 (2.00–5.00)	3.00 (2.00–5.00)	3.00 (2.00–5.00)
BMI kg/m^2^, M (SD)	37.14 (6.43)	37.11 (6.00)	37.16 (6.87)	31.95 (7.76)	32.71 (8.41)	31.21 (7.00)
BMI < 35 kg/m^2^	108 (46.8%)	54 (46.96%)	54 (46.55%)	167 (72.29%)	76 (66.09%)	91 (78.45%)
BMI < 27 kg/m^2^	1 (0.43%)	1 (0.87%)	0 (0.00%)	66 (28.57%)	32 (27.83%)	34 (29.31%)
Weight, kg, M (SD)	106.55 (19.41)	106.20 (19.03)	106.90 (19.84)	97.57 (28.12)	100.23 (30.06)	94.93 (25.91)
Daily caloric intake, kcal, M (SD)^d^	2142.88 (733.43)	2182.66 (730.00)	2103.10 (737.88)	2212.09 (798.38)	2234.57 (875.47)	2189.61 (716.34)
Daily steps, M (SD)^e^	8114.40 (3512.68)	8023.37 (3412.32)	8203.70 (3622.56)	--	--	--
Relationship closeness^f^	6.37 (0.64)	6.38 (0.62)	6.37 (0.66)	6.27 (0.68)	6.28 (0.67)	6.25 (0.70)
Inclusion of other in self-manage eating	3.94 (1.30)	3.97 (1.39)	3.91 (1.22)	3.93 (1.42)	4.01 (1.37)	3.85 (1.48)
Couple efficacy for diet	8.15 (1.36)	8.14 (1.42)	8.16 (1.30)	7.92 (1.59)	7.98 (1.55)	7.87 (1.63)
Outcome efficacy for diet	8.82 (1.09)	8.87 (1.11)	8.77 (1.07)	8.49 (1.40)	8.57 (1.27)	8.42 (1.52)
Communal coping for diet	1.59 (0.79)	1.59 (0.86)	1.58 (0.73)	1.67 (0.73)	1.65 (0.73)	1.68 (0.88)
Social support for healthy eating^g^	2.93 (0.75)	2.98 (0.73)	2.88 (0.77)	2.90 (0.72)	2.90 (0.72)	2.83 (0.78)
Sabotage for healthy eating^g^	2.65 (0.73)	2.64 (0.74)	2.65 (0.74)	2.65 (0.64)	2.65 (0.64)	2.65 (0.73)
Inclusion of other in self-physical activity	3.31 (1.47)	3.27 (1.52)	3.34 (1.42)	3.48 (1.49)	3.42 (1.47)	3.54 (1.52)
Couple efficacy for physical activity	7.97 (1.49)	7.96 (1.51)	7.97 (1.48)	7.86 (1.73)	7.94 (1.69)	7.78 (1.76)
Outcome efficacy for physical activity	8.36 (1.29)	8.45 (1.25)	8.27 (1.33)	8.15 (1.59)	8.25 (1.51)	8.05 (1.66)
Communal coping for physical activity	1.40 (0.88)	1.36 (0.90)	1.44 (0.86)	1.40 (0.81)	1.42 (0.85)	1.39 (0.77)
Social support for physical activity^h^	2.75 (0.78)	2.78 (0.81)	2.73 (0.76)	2.73 (0.80)	2.73 (0.80)	2.74 (0.81)
Sabotage for physical activity^h^	2.66 (0.77)	2.60 (0.74)	2.72 (0.80)	2.75 (0.74)	2.75 (0.74)	2.70 (0.74)

a All numbers are n(%) unless indicated otherwise.

b Partnership identities are based on gender identity.

c Sex assigned at birth was used for stratification of index participants and partners.

d Daily caloric intake is estimated based on responses to ASA24.

e Daily steps were measured by Fitbit accelerometer. Due to financial constraints, Fitbits were not provided to partners.

f Relationship closeness was measured with the 11-item Unidimensional Relationship Closeness Scale.

g Social support and sabotage for healthy eating were measured with items developed by Ball and Crawford.

h Social support and sabotage for physical activity were measured with items developed by Ball and Crawford.

Cronbach alphas for (sub)scales were: Unidimensional Relationship Closeness Scale (α = 0.91); couple efficacy for diet (α = 0.84); outcome efficacy for diet (α = 0.82); communal coping for diet (α = 0.42); social support for healthy eating (α = 0.64); sabotage for healthy eating (α = 0.38); couple efficacy for physical activity (α = 0.89); outcome efficacy for physical activity (α = 0.89); communal coping for physical activity (α = 0.61); social support for physical activity (α = 0.64); sabotage for physical activity (α = 0.38).

## Data Availability

The dataset generated during the current study is not publicly available but is available from the corresponding author on reasonable request.

## Abbreviations

AE: Adverse event
ASA24: Automated Self-Administered 24-Hour Dietary Assessment Tool
BMI: Body mass index
CBCT: Cognitive Behavioral Couples Therapy
LME: Linear Mixed Effects
REDCap: Research Electronic Data Capture
RD: Registered Dietician
SAE: Serious Adverse Event
SMART: Specific, Measurable, Actionable, Relevant, and Time-bound
